# WORKING WITH OR WITHOUT AGING SPECIALISTS: WHERE ARE THE GERONTOLOGISTS?

**DOI:** 10.1093/geroni/igz038.188

**Published:** 2019-11-08

**Authors:** Heidi H Ewen

**Affiliations:** University of Indianapolis, University of Georgia, Indianapolis, Indiana, United States

## Abstract

One question asked by generations of gerontology doctoral students is what types of employment can be secured after completing the PhD in Gerontology. The Gerontology Education Longitudinal Study (GELS) has surveyed graduate students and alumni of the various doctoral programs in order to understand the career trajectories of graduates. Of 102 alumni surveyed in 2014 (42% response rate), the majority (60%) were not working with other people who had degrees in gerontology, yet 51% report working with at least some people who have experience with aging and older adults. On the job, graduates say that their duties require knowledge and mastery of public policy issues, health and medical aspects related to aging, and psychological theories. As aging experts, it is inherent in their work to combat ageism and reduce age-related stereotypes. As such, gerontologists are using substantive content expertise within their careers and serving as experts in aging.

